# Nutrient content of fish powder from low value fish and fish byproducts

**DOI:** 10.1002/fsn3.402

**Published:** 2016-07-05

**Authors:** Lawrence Abbey, Mary Glover‐Amengor, Margaret O. Atikpo, Amy Atter, Jogeir Toppe

**Affiliations:** ^1^ CSIR‐Food Research InstituteAccraGhana; ^2^ Food and Agriculture Organisation (FAO)RomeItaly

**Keywords:** Burrito, fish powder, micronutrient, tuna, underutilized fish

## Abstract

Consuming small‐sized fish species whole, and bones of large fish could contribute significantly to reducing the level of micronutrient and protein malnutrition. These fish products are more affordable and could therefore meet the needs of poor, vulnerable groups, particularly in rural and urban areas where limited economic resources prevent dietary diversity. The objectives of the study were to produce fish powder from dried edible byproducts from fish processing factories, an underutilized fish species, burrito and to determine the physical, micromineral, macronutrient and microbiological quality of the dried fish powder. Edible fish processing byproducts and an underutilized fish, burrito (*Brachydeuterus auritus*) were cleaned thoroughly and dried with a Council for Scientific and Industrial Research‐Food Research Institute (CSIR‐FRI) gas‐fuelled oven at 55°C for 8 h or until dried. The dried products were milled into powder, and packaged into polythene bags. Proximate analysis of the fish powder was done Official Methods of Analysis (AOAC) methods. Minerals and heavy metals in the fish powder were determined by atomic absorption spectrophotometry (AAS). Microbiological quality was determined by Nordic Committee on Food Analysis Method (NMLK) methods. Tuna trimmings contained 80.71 g/100 g protein, whereas burrito contained 70.40 g/100 g protein. Concentrations of cadmium, arsenic, and mercury varied from <1.00 to 1 mg/kg. Lead was found at 0.04 mg/100 g in tuna frames and gills only. All fish byproducts contained high levels of iron, for example, trimmings contained 16.58 mg/100 g, whereas tuna frames and gills also contained 16.82 and 19.54 mg/100 g, respectively. Burrito contained 8.92 mg/100 g. Zinc levels also ranged from 0.41 mg/100 g in tuna trimmings to 1.88 mg/100 g in tuna gills. The powdered samples according to the standards set by Ghana Standard Authority, were acceptable. Consuming small‐ sized fish species whole, and bones of large fish could contribute significantly to reducing the level of micronutrient and protein malnutrition. These are more affordable and could therefore meet the needs of poor, vulnerable groups.

## Introduction

More than 2 billion people are affected by micronutrient deficiency (WHO 2001), a condition often referred to as “hidden hunger.” Micronutrient deficiency is in particular prevalent in poor rural and urban areas where limited economic resources prevent diversity in diets.

The most common micronutrient deficiencies are connected to low dietary intakes of vitamin A, iron, and iodine (Allen et al. 2006). However, other more neglected micronutrient deficiencies are due to nonavailability of selenium, zinc, and calcium in the diet which significantly affect the health of individuals (Capon and Smith 1982). It has been reported that zinc deficiency contributes to the death of 800,000 children globally per year, whereas rickets caused by calcium deficiency is gaining more attention than before (Hagan et al. 2010). Omega‐3 fatty acid deficiency is also important, but not measured in this study.

Fish products are considered a good source of many micronutrients of significance. Fish is also a cheaper and preferred source of animal protein (Ashitey and Flake 2010); Gordon et al. (2011). The levels of most of the minerals are found in high amounts in fish bones. However, apart from eating small‐sized fish species whole (with the bones inclusive), consumption of fish bones of larger fish is rarely practiced. An increased use of seafood, including bones, could contribute significantly to reducing the level of micronutrients and protein malnutrition (Toppe 2014). Many vulnerable groups cannot afford to buy seafood products, especially in areas where seafood is not available. A solution to the economic and logistic challenges in increasing fish consumption among the poor will be essential in order to make seafood accessible and affordable in micronutrient deficient areas. High‐quality fish products from underutilized small pelagic fishes and edible fish processing byproducts that can easily be stored and transported should be considered as supplement to diets in such areas. The product should have a potential of being easily introduced into local diets and acceptable by the indigenous population.

Small pelagic fish are among the most affordable and healthy fish. Two meals a week of most carps, for example, will be adequate, and no fish oil is needed in their feed in order to become a good source of beneficial omega‐3 oils (Toppe 2014). Consuming one hundred grams of small pelagic fish such as sardines or anchovies once a week will more than cover the needs of omega‐3s for a person. Fish products should be processed from low cost high‐quality fish. Improved utilization of existing fishery resources could also play a more important role in meeting the increasing demand of valuable nutrients from the aquatic environment. Reducing postharvest losses, estimated at more than 10% in volume and up to 30% in value, could release millions of tons of healthful fish products for consumption (Toppe 2014). Byproducts as a result of processing represent in many cases more than 50% of the fish being processed. These byproducts are in many cases low‐cost products, but with high nutritional value (Toppe 2014); Kabahenda et al. (2011).

This study is part of an FAO/CSIR‐FRI collaborative project, 2015, that aims at developing low‐cost nutrient dense fish products for National School Feeding Initiatives, utilizing low value (underutilized) fish and edible fish byproducts; the project further aims at using small‐ and medium‐scale processing and preservation methods that would stabilize nutritional value and ensure food safety.

## Specific Objectives of Study

1. To produce fish powder from dried edible byproducts from fish processing factories.
2. To produce fish powder from dried underutilized fish, burrito.
3. To determine the physical, micromineral, macronutrient and microbiological quality of the dried fish powder.

## Materials and Methods

### Production of tuna byproducts powder (trimmings, gills and frames)

Tuna byproducts, tightly packaged in clear polypropylene bags and frozen were obtained three consecutive times from Cosmo Seafoods Company in Tema, in April and May, 2015, and transported in an ice‐chest to the CSIR‐Food Research Institute.

The byproducts were washed, arranged on trays in a mechanical dryer, and dried to moisture contents of 4.8% (fish trimmings), 8.9% (frames), and 6.8% (fish gills) in a CSIR gas‐fuelled dryer. These were milled into powder using a hammer mill with a 250 *μ*m mesh sieve (Model 160B; Jacobson Machinery Works, Minneapolis). The composite fish powdered products were packaged into polypropylene bags and stored at −18**°**C until ready for use.

**Figure 1:**
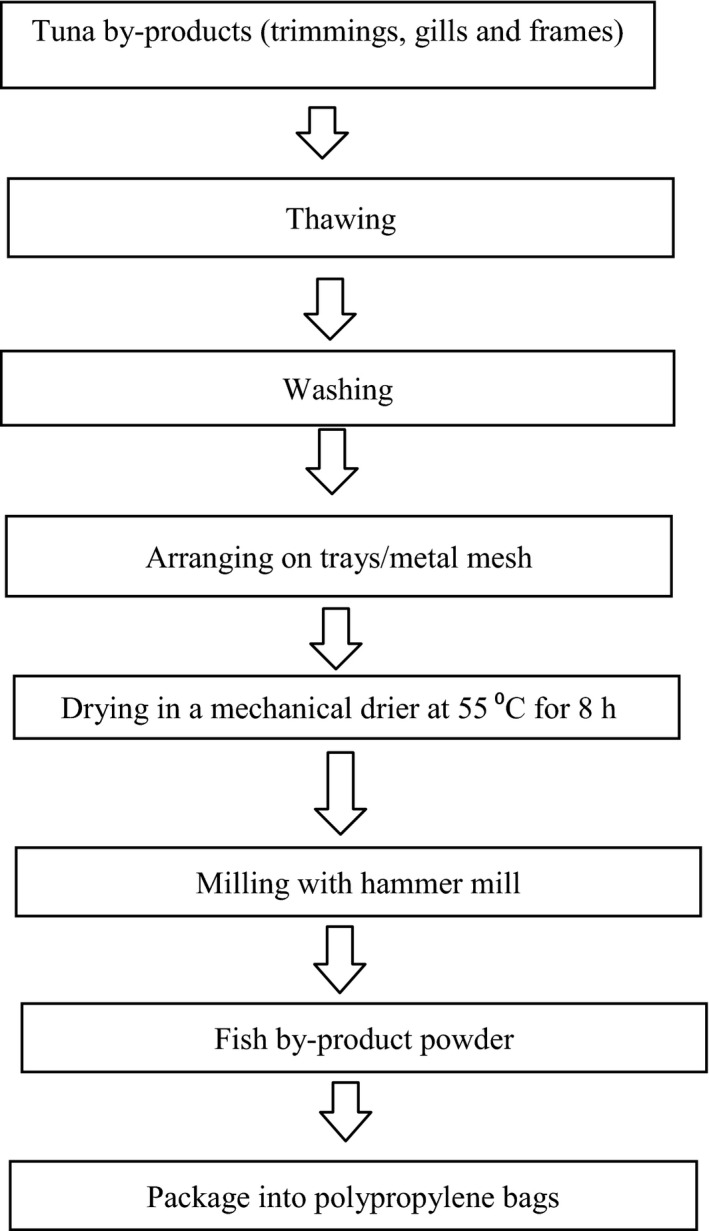
Flow chart for fish byproducts powder production.

The process flow chart for the production of tuna byproducts powder is shown in Figure 1.

### Production of burrito fish (*Brachydeuterus auritus*) powder

#### Materials

In April and May, 2015, fresh burrito fish *(Brachydeuterus auritus)* was purchased from the Tema Fish Market and iced. Samples were purchased three times at 2 week intervals. The fish was subsequently frozen and held at between −15 and −17**°**C until ready to use, when the fish was thawed.

#### Methods

Descaled, degutted, and washed burrito fishes *(Brachydeuterus auritus)* were arranged on perforated trays and mechanically dried in a CSIR gas‐fuelled dryer at 55**°**C for 8 h or until well dried with a moisture content of about 6.9%. The dried fish was milled using the Jacobson Hammer mill (Model 160B; Jacobson Machinery Works, Minneapolis) with a 250 *μ*m mesh sieve and packaged into polypropylene pouches (24 cm × 14 cm) with a gauge of 49.24 mil. The composite powders from the three replications were stored at −18**°**C until ready for use.

**Figure 2:**
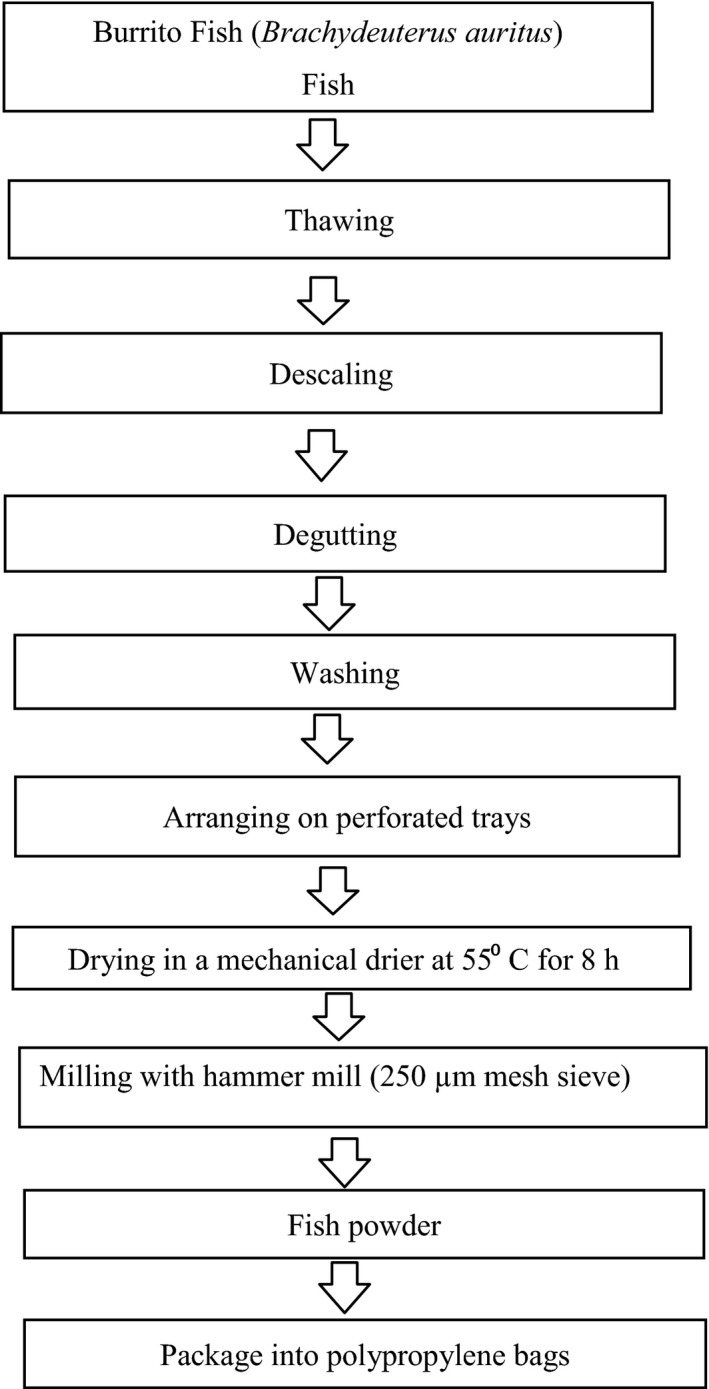
Flow chart for burrito fish powder production.

The production process flow chart of burrito fish powder is shown in Figure 2.

### Physicochemical analysis

Moisture content of the dried fish products was determined by AOAC (2000a). Water activity was determined using the Hygrolab water activity meter. About 15 g of fish powder was put into the sample container. This was then put in the sample chamber and the measuring head was put on it. It was run and the value read. Three readings were taken and the average value computed. Ash was determined by AOAC (2000b). Iron, phosphorus, and calcium were determined using 2,2‐bipyridyl colorimetric, Molybdenum blue colorimetric, and Permanganate titration methods, respectively (James 1990). Fat was determined by AOAC (2000c). Energy was determined by atwater factor (Pearson's composition and analysis of foods 1995). Protein was determined by Kjedahl method (AOAC 1990). Heavy metals were determined by AOAC (2005). Tests were run in triplicates and values recorded as means.

### Microbiological analysis

The microbial safety of the powdered burrito and the fish byproducts were determined by assaying for various indicator and pathogenic microorganisms using ISO and NMKL methods. These microorganisms included aerobic mesophiles (Nordic Committee on Food Analysis Method 1999a), yeast and molds (International Standards Organization Method 1987), coliform bacteria (Nordic Committee on Food Analysis Method 2004), *E. coli* (Nordic Committee on Food Analysis Method 2005), *Enterococcus* (Nordic Committee on Food Analysis Method 2000)*, Staphylococcus aureus* (Nordic Committee on Food Analysis Method 2003)*, Bacillus cereus* (Nordic Committee on Food Analysis Method 2010)*, Clostridium perfringens* (International Standards Organization Method 2004)*, Vibrio cholera* (International Standards Organization Method 2007), and *Salmonella typhimurium* (Nordic Committee on Food Analysis Method 1999b).

### Statistical analysis

Statistical analysis was done using Excel spreadsheet and Statistical package for social scientists (SPSS) version 21 (SPSS Inc, Chicago, USA). Analysis of variance (ANOVA) and Duncan test were used to test significant differences between samples (*P* < 0.05).

## Results

Tuna trimmings contained 80.71 g/100 g protein; burrito 70.40 g/100 g protein (Table 1). Trimmings contained 16.58 mg/100 g iron; tuna frames and gills contained 16.82 mg/100 g and 19.54 mg/100 g iron, respectively. Burrito contained 8.92 mg/100 g iron. Zinc levels also ranged from 0.41 mg/100 g in tuna trimmings to 1.88 mg/100 g in tuna gills. Calcium content was1066.50 mg/100 g in tuna trimmings, 13184.30 mg/100 g in tuna frames, 15469.30 mg/100 g in tuna gills, and 2586.63 mg/100 g in burrito.

**Table 1:** Proximate and Chemical results of dried powder prepared tuna processing byproducts and burrito

Parameter	Tuna trimmings	Tuna frames	Tuna gills	Burrito
Moisture (g/100 g)	4.8 ± 0. 0.13^b^	8.4 ± 0.10^d^	6.8 ± 0.24^c^	3.5 ± 0.03^a^
Water Activity (a_w_)	0.6 ± 0.01^a^	0.65 ± 0.3^a^	0.62 ± 0.01^a^	0.6 ± 0.00^a^
Ash (g/100 g)	3.4 ± 0.78^a^	44.11 ± 0.03^d^	42.99 ± 0.05^c^	14.0 ± 0.13^b^
Fat (g/100 g)	5.7 ± 0.12^c^	11.3 ± 0.03^a^	4.5 ± 37^b^	11.1 ± 14^a^
Protein (g/100 g)	80.71 ± 0.16^d^	28.66 ± 0.16^a^	38.29 ± 0.20^b^	70.4 ± 0.11^c^
Carbohydrate (including fiber) (g/100 g)	5.39 ± 0.24^c^	7.53 ± 0.09^a^	7.42 ± 0.37^a^	1.0 ± 35^b^
Energy (Kcal/100 g)	395.7 ± 1.45^d^	242.5 ± 0.14^b^	223.3 ± 1.11^a^	381.5 ± 3.25^c^
Phosphorus (mg/100 g)	600.9 ± 26.66^b^	1010.2 ± 12.16^c^	1071,8 ± 3.14^d^	93.71 ± 4.99^a^
Calcium (mg/100 g)	1066.5 ± 24.20^a^	13184.3 ± 56.53^c^	15469.3 ± 4.80^d^	2586.63 ± 4.26^b^

Results are presented as means and standard deviations. Analysis of variance (ANOVA) and Duncan test were used to significant differences between samples (*P* < 0.05). Means with the same superscripts are not significantly different from each other.

The concentrations of lead, copper, iron, zinc, manganese, arsenic, cadmium, and mercury are presented in Table 2. The results of the analysis indicate that the concentrations of cadmium, arsenic, and mercury varied from <1.00 mg/kg to 1.00 mg/kg for all the fish types. Lead was found at 0.04 mg/100 g in tuna frames and gills only.

**Table 2:** Heavy metals content of dried powder prepared from Tuna processing byproducts and Burrito

Parameter	Tuna trimmings	Tuna frames	Tuna gills	Burrito
Lead (mg/100 g)	Not detected^a^	0.04 ± ^b^	0.04 ± ^b^	Not Detected^a^
Copper	0.06 ± 0.00^a^	0.250.14^c^	0.140.02^b^	0.08 ± 0.02^a^
Iron (mg/100 g)	16.58 ± 0.04^b^	16.82 ± 0.13^b^	19.54 ± 0.03^b^	8.92 ± 0.56^a^
Zinc (mg/100 g)	0.41 ± 0.21^a^	0.59 ± 0.22^a^	1.88 ± 0.13^b^	0.67 ± 0.11^a^
Manganese (mg/100 g)	0.11 ± ^d^	0.76 ± ^c^	1.03 ± ^b^	1.56 ± ^a^
Arsenic (mg/kg)	1.00 ± ^a^	<1.00 ± ^a^	1.00 ± ^a^	<1.00 ± ^a^
Mercury (mg/kg)	<1.00 ± ^a^	<1.00 ±^ a^	1.00 ±^ a^	<1.00 ± ^a^
Cadmium (mg/kg)	<1.00 ± ^a^	<1.00 ± ^a^	<1.00 ± ^a^	<1.00 ± ^a^

Results are presented as means and standard deviations. Analysis of variance (ANOVA) and Duncan test were used to significant differences between samples (*P* < 0.05). Means with the same superscripts are not significantly different from each other.

### Microbiological analyses of dried powders of burrito and edible fish by products

**Figure 3:**
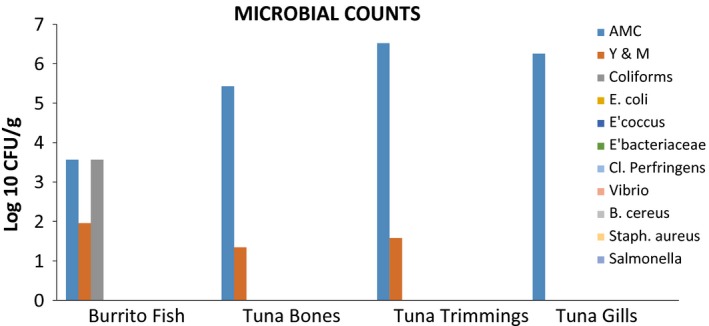
Microbial counts on the fish and fish by products.

The microbiological analyses of dried powders of burrito and edible fish by products are shown in Figure 3.

## Discussion

### Nutrient content of fish powders

Tuna trimmings contained 80.71 g/100 g protein; burrito 70.40 g/100 g protein, values high enough to quality them as good sources of protein. All fish byproducts contained high and almost equal levels of iron, for example, trimmings contained 16.58 mg/100 g; tuna frames and gills 16.82 mg/100 g and 19.54 mg/100 g, respectively. Burrito also contained 8.92 mg/100 g iron. Kabahenda et al. (2011) also assessed micronutrient and protein levels in low value fish and processing byproducts in fish from Lake Victoria region and found crude protein in the range of 47.9–58.8% in *mukene*, a low value fish. *Mukene* also contained 8.18–10.91 mg/100 g iron and 4.07–10.25 mg/100 g zinc on dry basis, suggesting that consuming low value fishes could improve protein and micronutrient levels of low socioeconomic class. An earlier work done by Glover‐Amengor et al. (2012), found protein levels ranging from 44.83% to 72.29% in some dried underutilized fish species.

The levels of protein and iron in burrito and tuna byproducts in this study are comparable to the levels in *mukene*, flying gurnard, *woevi,* and anchovies Glover‐Amengor et al. (2012) indicating that the fish powders could serve as good sources of protein and iron for the low income group.

Recommended dietary intake (RDI) of iron for children 9–13 years per day is 8 mg. Hence consumption of 50 g of tuna byproducts and 100 g of burrito powder could adequately meet the iron needs of children in these age brackets Zinc levels (0.41 mg/100 g in tuna trimmings to 1.88 mg/100 g in tuna gills), though not adequate to meet the RDI of 8 mg per day, could also supplement other dietary sources.

The results of the analysis of heavy metals indicated that the concentrations of cadmium, arsenic, and mercury varied from <1.001 mg/kg to 1 mg/kg (<0.10 to 0.10 mg/100 g). Lead was found at 0.04 mg/100 g in the tuna frames and gills only. Because of the known toxicity of mercury, lead, and cadmium, The Joint Food and Agriculture Organization/World Health Organization (FAO/WHO) Expert Committee on Food Additives suggested a provisional tolerable intake of 0.007 mg/kg body weight for cadmium per week from sea fish; 0.3 mg/kg body weight per week for mercury and lead, a weekly intake of 3.0 mg/kg body weight (FAO/WHO 2004).

These underutilized fish species and fish processing by products could be easily dried with gas and solar driers or smoked, milled and stored for use in homes and also on National school feeding programs. Whereas waste could be avoided in fish processing factories through the up‐take of byproducts, people of low income levels could also be nourished through the consumption of these processed products. Fishermen also stand to increase their income levels through this value chain as demand for their catch will increase.

### Microbiological safety of fish powders

Although the Ghanaian Standards usually request for levels of aerobic mesophiles, *Staphylococcus aureus,* and *E. coli* or fecal coliform bacteria, this work tested for other pathogens as well to ascertain its safety. The population of aerobic mesophiles ranged between 10^5^ and 10^6^ colony‐forming unit (CFU/g) for the fish byproducts powder, whereas the burrito fish powders were at 10^6^ CFU/g. The population of yeast and moulds were lower at 10^2^ CFU/g and below. Coliform bacteria of a population of 10^2^ CFU/g were recorded from the burrito fish powder. The pathogens *E. coli*,* Enterococcus, Enterobacteriaceae, Staphylococcus aureus, Bacillus cereus, Clostridium perfringens, Vibrio cholerae,* and *Salmonella* were not detected in any of the powdered samples. Species of fungi have generally been associated with fish samples; however, the mechanical drying process used for these samples was effective in their inactivation thereby recording acceptable results as shown in Figure 3. Moreover, the fish powder will be incorporated while cooking the food, so heat during cooking will destroy all microbes present.

## Conclusion

The protein and iron content of all fish powders were high, and these could therefore serve as good and affordable sources of these nutrients for the poor, vulnerable groups. The powdered samples according to Ghana standards (2013) were acceptable. The burrito and tuna‐by products powders were therefore microbiologically safe for inclusion in human diets and this will improve food security in Ghana.

## Conflict of Interest

None declared.
